# Chemotherapy reduces PARP1 in cancers of the ovary: implications for future clinical trials involving PARP inhibitors

**DOI:** 10.1186/s12916-015-0454-9

**Published:** 2015-09-09

**Authors:** Maud Marques, Marie-Claude Beauchamp, Hubert Fleury, Ido Laskov, Sun Qiang, Manuela Pelmus, Diane Provencher, Anne-Marie Mes-Masson, Walter H. Gotlieb, Michael Witcher

**Affiliations:** Lady Davis Institute for Medical Research, Jewish General Hospital, 3755 Côte Ste, Catherine Road, Montréal, QC H3T 1E2 Canada; Centre de recherche du Centre hospitalier de l’Université de Montréal/Institut du cancer de Montréal, Montréal, Canada; Division of Gynecologic Oncology, Jewish General Hospital, McGill University, Montréal, Canada; Division of Pathology, Jewish General Hospital, Montréal, Canada; Department of Obstetric-Gynecology, Université de Montréal, Montreal, Canada; Department of Medicine, Université de Montréal, Montreal, Canada

**Keywords:** Chemotherapy, PARP inhibitors, PARP1, ovarian cancer

## Abstract

**Background:**

PARP inhibitors have shown promising clinical results in cancer patients carrying *BRCA1/2* mutations. Their clinical efficacy could logically be influenced by PARP1 protein levels in patient tumors.

**Methods:**

We screened three cohorts of patients with ovarian cancer, totaling 313 samples, and evaluated PARP1 protein expression by immunohistochemistry with further validation by western blotting.

**Results:**

We observed that up to 60 % of tumors showed little PARP1 protein expression. In serous ovarian tumors, comparing intratumoral PARP1 expression between chemo-naïve and post-chemotherapy patients revealed a decrease in intratumoral PARP1 following chemotherapy in all three cohorts (immunohistochemistry: *p* < 0.001, n = 239; western blot: *p* = 0.012, n = 74). The findings were further confirmed in a selection of matched samples from the same patients before and after chemotherapy.

**Conclusion:**

Our data suggest that patients should be screened for PARP1 expression prior to therapy with PARP inhibitors. Further, the observed reduction of intratumoral PARP1 post-chemotherapy suggests that treating chemo-naïve patients with PARP inhibitors prior to the administration of chemotherapy, or concurrently, might increase the responsiveness to PARP1 inhibition. Thus, a change in the timing of PARP inhibitor administration may be warranted for future clinical trials.

**Electronic supplementary material:**

The online version of this article (doi:10.1186/s12916-015-0454-9) contains supplementary material, which is available to authorized users.

## Background

Unlike breast cancer, the overall survival of patients with ovarian cancer has shown only slight improvement over the past 30 years [1], with high-grade serous cancers (HGSC) of the ovary being the most deadly gynecologic cancer. While many patients with HGSC respond to first-line chemotherapy, recurrence and drug resistance occur in the vast majority of cases [2]. Identification of key biomarkers or predictors for response to one or a combination of specific antitumor drugs seems essential.

Over the past 5 years, clinical trials using various PARP inhibitors on patients with *BRCA1/2* mutations [3, 4] have shown promising results [5]. Inherent defects in the homologous recombination DNA repair pathway in *BRCA*-deficient tumors coupled with the inhibition of PARP1 lead to a synthetic lethality culminating in cancer cell death [6]. It is estimated that almost half of patients with HGSC bear defects in homologous recombination [7], spurring the onset of clinical trials to test PARP inhibitors in this population.

The reported response rate for *BRCA*-deficient HGSC in the ovary treated with PARP inhibitors ranges between 25 and 53 % [5, 8–12]. In these published studies, enrolled patients had received several cycles of adjuvant chemotherapy prior to PARP inhibitors.

In this study, we evaluated the effect of chemotherapy on PARP1 expression in solid tumors.

## Methods

### Patients

Neo-adjuvant chemotherapy consisted of three to four combined courses of a platinum-based agent (carboplatin) and paclitaxel given prior to surgery. Changes to this protocol were made depending on the patient's condition, response, compliance, and side effects. The patients were considered responsive to this regimen if recurrence occurred more than 6 months after the last chemotherapy treatment.

#### Training cohort: immunohistochemistry (IHC) analysis

Tumor samples were obtained from 69 patients who had undergone surgery and chemotherapy in the Division of Gynecologic Oncology of McGill University’s Jewish General Hospital from 2006 to 2014 (Table 1). Diagnosis was based on final pathology, determined by an independent gynecologic pathologist. The tumors were staged according to the International Federation of Gynecology and Obstetrics (FIGO) stage and histologically classified and graded according to the World Health Organization grade. Tissue selection criteria for this study were based on chemotherapy-naïve and chemotherapy-treated patients.

**Table 1:** Training cohort: clinical characteristics of patients for immunohistochemistry experiment

Patients		Number (%)
Number		69
Age (years), mean (range)		61.9 (36–86)
Histology	Serous	69 (100)
Stage	I	6 (9)
	II	6 (9)
	III	51 (74)
	IV	5 (7)
Grade	1	2 (3)
	2	3 (4)
	3	57 (83)
Chemotherapy	Neo-adjuvant	35 (51)
	Adjuvant	32 (46)
Surgery	Not robotic	32 (46)
	Robotic	35 (51)
Residual disease		4 (6)
Response	Responsive	41 (59)
	Resistant	19 (28)
Recurrence	Number	41 (59)
	Mean time (range)	17.9 months (3–84)
Deceased	Number	21 (30.4)
	Mean time (range)	27.9 months(10–72)
Follow-up	Mean time (range)	36.6 months (7–111)

#### Validation cohort 1: Tissue microarray (TMA)

Tumor samples were obtained from 170 patients who had undergone surgery for ovarian cancer at the Department of Gynecologic Oncology Centre Hospitalier de l’Université de Montréal (Table 2). An independent pathologist reviewed tumor histopathology, grade, and chemotherapy status of each patient. Tissue selection criteria for this study were based on chemotherapy-naïve and chemotherapy-treated patients. Samples were collected between 1993 and 2014.

**Table 2:** Validation cohort 1: clinical characteristics of patients for tissue microarray experiment

Patients		Number (%)
Number		170
Age (years), mean (range)		52 (39–93)
Histology	Serous	150 (88.2)
	Non serous	20 (11.8)
Stage	I	7 (4.1)
	II	11 (6.4)
	III	129 (72.9)
	IV	21 (12.3)
Grade	1	6 (3.5)
	2	22 (12.9)
	3	136 (80)
Chemotherapy	Neo-adjuvant	85 (50)
	Adjuvant	85 (50)
Residual disease	≤1 cm	87 (51.2)
	≥1 cm	57 (33.5)
	Not determined	26 (15.3)
Response	Responsive	123 (72.3)
	Resistant	47 (27.7)
Recurrence	Number	128 (75.3)
	Mean time (range)	20 months (2–76)
Deceased	Number	105 (61.8)
	Mean time (range)	47 months (2–156)
Follow up	Mean time (range)	43 months (2–73)

#### Validation cohort 2: western blot analysis

Tissue samples were included from 74 patients (mean age 60.7, range 24–87) with ovarian cancer who had undergone surgery and chemotherapy in the Jewish General Hospital from 2004 to 2013 (Table 3). The patient’s course of treatment was determined by a team of gynecologic oncologists in accordance with the National Comprehensive Cancer Network guidelines.

**Table 3:** Validation cohort 2: clinical characteristics of patients for western blot experiment

Patients		Number (%)
Number		74
Age(y), mean(range)		60.7 (24–87)
Histology	Serous	48 (64.9)
	Non Serous	26 (35.1)
Stage	I	11 (16.4)
	II	5 (7.5)
	III	42 (62.7)
	IV	9 (13.4)
Grade	1	9 (16.9)
	2	5 (9.4)
	3	39 (73.6)
Chemotherapy	Neo-adjuvant	34 (46)
	Adjuvant	25 (34)
Surgery	Laparotomy	30 (46)
	Laparoscopy	2 (3)
	Robotic	33 (51)
Residual disease		8 (10.8)
Response	Responsive	33 (75)
	Resistant	11 (25)
Recurrence	Number	27 (36.5)
	Mean time (range)	9.6 months (3–29)
Deceased	Number	21 (31.3)
	Mean time (range)	26.5 months (4–88)
Follow-up	Mean time (range)	31.4 months (7–107)

#### Ethical approval

The research complied with all requirements of the Helsinki Declaration. Tissue samples were taken from the gynecologic oncology tumor bank and approved by the Jewish General Hospital (protocol#14-025). Ethic approval was obtained from the Jewish General Hospital Research Ethics Committee (protocol#03-041). All patients participating in this study gave informed written consent.

### PARP1 antibody validation

PARP1 antibody (Clone B-10, sc74470, Santa Cruz Biotechnology, Inc. Dallas, TX, USA) specificity was validated for both western blot and IHC (Additional file 1: Figure S1). Epithelial ovarian cancer cells OVCAR8 were infected using shctl or shPARP1. After 5 days, cells were collected and either fixed in formalin for IHC analysis or proteins were extracted for western blot analysis.

### Immunohistochemistry

Biopsies and surgical specimens were formalin-fixed at the Pathology Facility (Jewish General Hospital) for 12 h at room temperature, processed, and paraffin-embedded. IHC staining was performed at the Segal Cancer Center Research Pathology Facility as previously described [13]. Briefly, tissue samples were cut at 4 μm, placed on SuperFrost/Plus slides (Fisher Scientific, Pittsburgh, PA, USA), and dried overnight at 37 °C. The slides were then loaded onto the Discovery XT Autostainer (Ventana Medical Systems, Inc. Tucson, AZ, USA). All solutions used for automated IHC were from Ventana Medical Systems unless otherwise specified. Slides underwent de-paraffinization with the EZ PREP solution (Ref# 950–100), followed by heat-induced epitope retrieval with Cell Conditioning Solution 1, pH 8.0 (Ref# 950–224), in standard conditions (60 min at 95 °C). Immunostaining for PARP1 was performed online using a heat protocol. Briefly, mouse monoclonal anti-PARP1 (B-10, sc74470, Santa Cruz Biotechnology, Inc.) diluted at 1:100 in antibody diluent (Ref# 251–018) was manually applied for 32 min at 37 °C, then PARP1 protein was detected using Omnimap anti-Mouse HRP Kit (Ref# 760–4310) and ChromoMap-DAB (Ref# 760–159). Slides were counterstained with hematoxylin for 4 min; blued with Bluing Reagent for 4 min; removed from the autostainer; washed in warm soapy water (Dawn) dehydrated through graded alcohols; cleared in xylene; and mounted with Permount. The percentage of tumor cells stained was assessed by an experienced pathologist (MP, assisted by MM, M-CB and IL) and ranked as follow: 0 = no staining, 1 = <25 % staining, 2 = 25–50 %, 3 = >75 %. The intensity of the staining was ranked as follow: 0 = no staining, 1 = mild staining, 2 = moderate staining, 3 = strong staining. Scores for percentage of distribution and intensity were multiplied by each other to reach a final score ranging between 0 and 9 (intensity × distribution) for comparison analysis. For TMA analysis, the intensity of staining was evaluated by two people blinded to the identification of the cores. More than 80 % of similarity was observed between the two independent scores. Only tumor tissue was taken into account for the score calculation.

### Pre-post chemotherapy epithelial ovarian tumor tissue microarray

A gynecologic pathologist reviewed all samples pre-chemotherapy and post-chemotherapy to identify the grade and type of ovarian carcinoma. Areas of interest were marked on slides. Two cores of 0.6 mm for each tissue sample were arrayed onto one recipient paraffin block. This tissue array was composed of cores from 170 epithelial ovarian tumors (two cores per patient sample) and was built on two recipient blocks.

### Protein extraction and western blot analysis

In total, 100 unselected tumor samples from 74 patients (cohort 3) were analyzed, comprising 21 ascites cells pellet, 57 primary tumors, 18 omental tumors, and 4 other tumor tissues. Similar levels of PARP1 protein were observed between the tumor and the omentum isolated from the same patient at the time of surgery (data not shown). Snap-frozen tumor tissues were minced and lysed in lysis buffer (25 mM Tris∙HCl pH 7.6, 10 % glycerol, 420 mM NaCl, 2 mM MgCl_2_, 0.5 % NP-40, 0.5 % Triton X-100, 1 mM EDTA, protease inhibitor) on ice. Briefly, clarified protein lysates (50 μg) were resolved on 6 % and 10 % denaturing SDS-polyacrylamide gels, and transferred to nitrocellulose membranes, similar to as previously described [14]. After blocking in 5 % milk, membranes were probed with primary antibodies specific for BRCA1 (cat#OP92, Calbiochem, Gibbstown, NJ, USA), PARP1 (sc74470), and β-actin (Cell Signaling Technology, Whitby, Ontario, Canada). Immunoblotted proteins were visualized using horseradish peroxidase-conjugated secondary antibodies and antigen-antibody complexes were detected using the ECL system (Bio-Rad Laboratories, Inc. Hercules, CA, USA).

### Statistical analysis

Statistical analysis was performed using SPSS (version 22) and R (version 3.1.0). The data were assessed as nonparametric data. A χ^2^ test was used to analyze the effect of chemotherapy on PARP1 and BRCA1 protein level [15]. We used a two-tailed Mann–Whitney U test to analyze dependency between treatment and PARP1 protein quantity [16]. A two-tailed Wilcoxon signed ranks test was used to evaluate this phenomenon in the same patient before and after chemotherapeutic treatment [15]. Differences with a *p*-value < 0.05 were considered statistically significant.

## Results and discussion

**Fig. 1:**
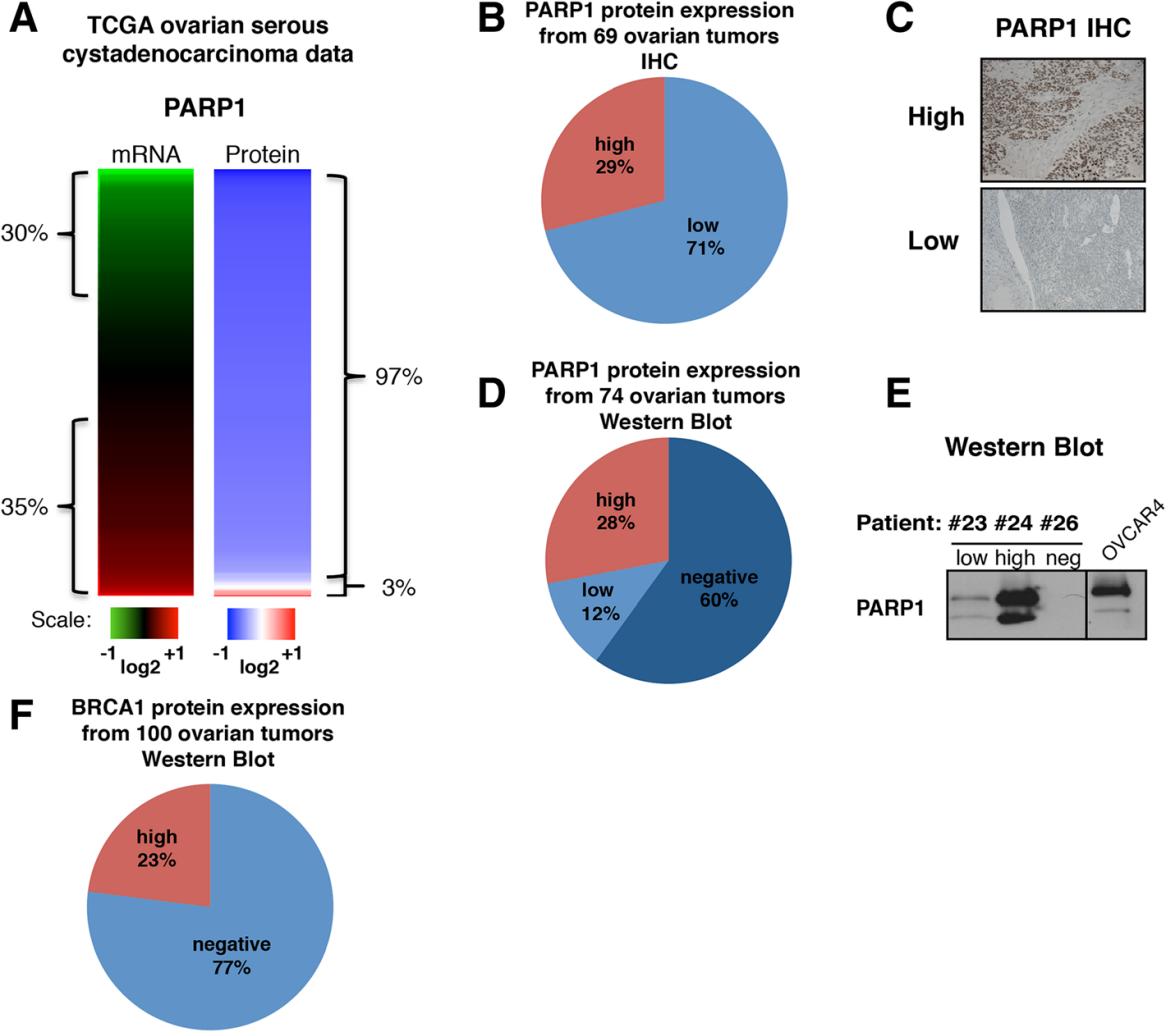
Low PARP1 protein expression in ovarian tumors. **a** Analysis of The Cancer Genome Atlas (*TCGA*) ovarian cystadenocarcinoma database for PARP1 mRNA (n = 570) and protein (n = 412) expression levels. Data were used to generate a heat map with *red* representing high expression level and *green* low expression level. **b** Pie chart representing PARP1 protein level as determined by immunohistochemistry (*IHC*) in 69 HGSC samples (training cohort). **c** PARP1 representative IHC for each expression category (low and high). **d** Pie chart distribution of PARP1 in 100 unselected ovarian tumors from 74 patients (validation cohort 2, several patients had multiple tumors examined) evaluated by western blotting. **e** PARP1 representative western blot for each expression category (low, high, and negative); OVCAR4 protein extract was used as positive control. **f** Pie chart representing BRCA1 protein level distribution from 74 patients (100 tumors, validation cohort 2) evaluated by western blotting

**Fig. 2:**
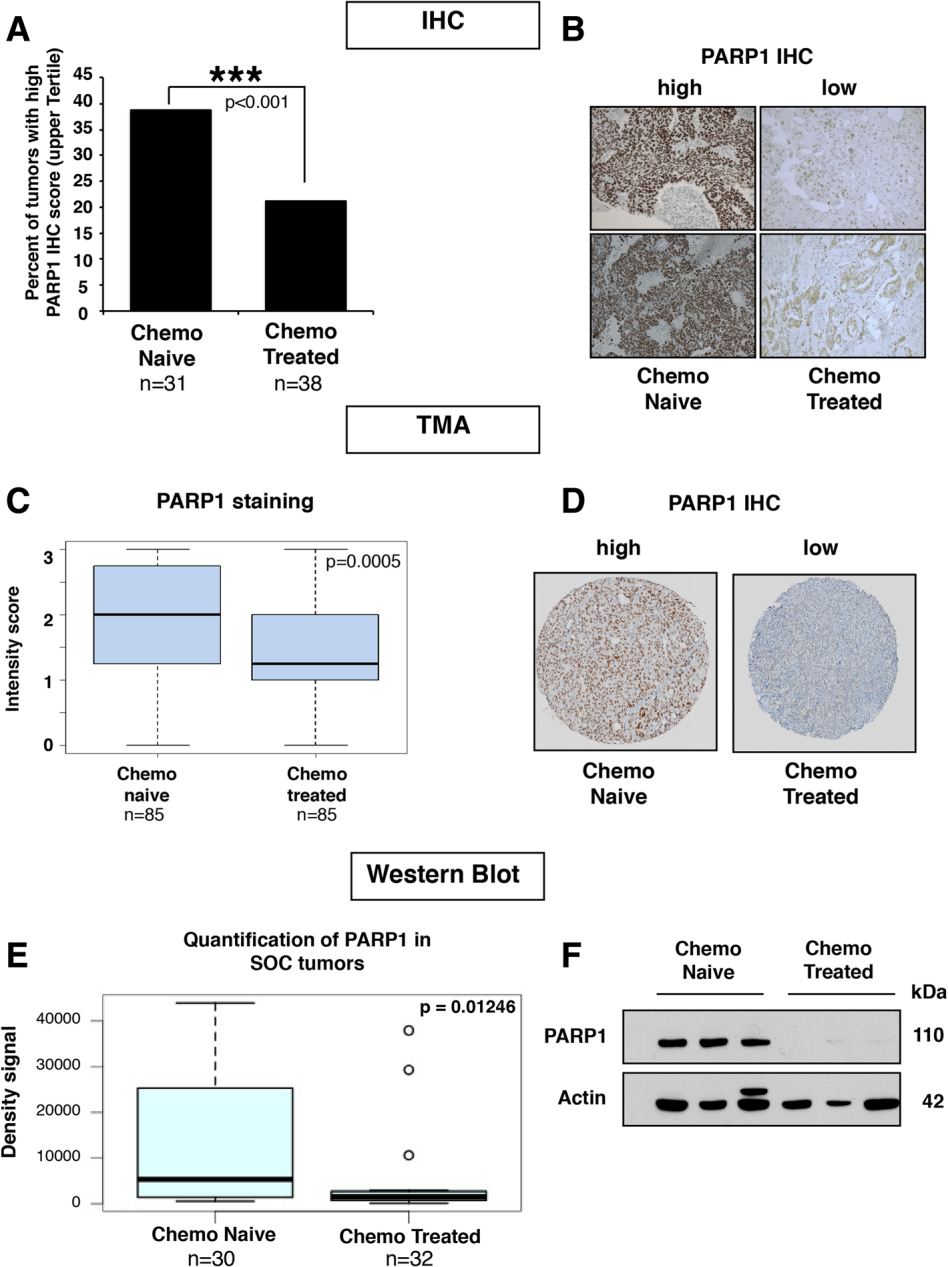
Chemo-treated tumors have lower levels of PARP1 protein than chemo-naïve tumors. **a** We used 69 HGSC tumors for immunohistochemistry (*IHC*) staining of PARP1 (training cohort). Each slide score is the product of the percentage of stained tumors cells and the intensity of the staining as described in the “Methods” section. The bar graph represents the percentage of slides with scores in the upper tertile from chemo-naïve and chemo-treated samples. Chi-square test *p* < 0.001. **b** Representative photos of PARP1 IHC from two chemo-naïve tumors and two chemo-treated tumors (20×). **c** Boxplot showing compilation of PARP1 intensity of staining score for the tissue microarray (*TMA*). The *p*-value tested whether there was a significant difference in PARP1 staining intensity between the chemo-naïve and chemo-treated tumors and was calculated using a two-tailed Mann–Whitney test. **d** Representative TMA cores stain with PARP1 antibody. **e** PARP1 protein levels in 62 serous ovarian cancer (*SOC*) tumors (30 chemo-naïve and 32 chemo-treated) were quantified with ImageJ and the density signals obtained were used to generate a boxplot. Wilcox Mann–Whitney test gave *p* = 0.01246. **f** Representative western blots of PARP1 in three chemo-naïve tumors and three chemo-treated tumors from cohort shown in **e**

We first analyzed gene expression (n = 570) and reverse-phase protein analysis (n = 412) data made publically available from The Cancer Genome Atlas (TCGA) website for HGSC and found low PARP1 protein levels in a majority of the tumors (Fig. 1), consistent with previous findings in breast tumors [17]. These data prompted us to evaluate PARP1 protein levels in a set of 69 HGSC by IHC (training cohort). Of these, 71 % were characterized as having low PARP1 expression (Fig. 1b,c). This data was further validated using western blotting of tumors from 74 unselected patients with ovarian cancer (validation cohort 2). PARP1 protein expression was undetectable in 60 % of the tumors analyzed (Fig. 1d,e), similar to our IHC data and previously published studies [18, 19]. Of the 100 tumors we analyzed from the 74-patient cohort by western blotting, we further observed 77 % of them had undetectable BRCA1 protein levels (Fig. 1f), and found the same ratio of PARP1-expressing and BRCA1-expressing tumors as seen in previous studies [18, 19]. This suggests that samples within this cohort are highly representative of HGSC cohorts examined by other groups. Because of the increasing use of neo-adjuvant therapy, we had the opportunity to evaluate PARP1 expression in cancers before and after chemotherapy and evaluate whether exposure to chemotherapy might play a role in the levels of PARP1 in ovarian tumor. We selected tumor samples from patients with HGSC and compared PARP1 protein levels between chemo-naïve and chemo-treated tumors with standard chemotherapy (carboplatin + taxol) in three patient cohorts: a training cohort (IHC, 69 HGSC samples), validation cohort 1 (IHC, 170 HGSC samples), and validation cohort 2 (western blotting, 74 patients, 62 HGSC samples). We found a significant reduction of PARP1 protein by IHC on the training cohort of 31 chemo-naïve and 38 chemo-treated HGSC (Fig. 2a and Table 1). The proportion of tumors with high PARP1 expression was halved in chemo-treated samples compared to the chemo-naïve tumors (Fig. 2a,b). We next validated these results using validation cohort 1 for a TMA (Fig. 2c,d and Table 2) and validation cohort 2 for western blotting (Fig. 2e,f and Table 3). In both groups, we found a significant reduction of PARP1 expression in the chemo-exposed tumors.

**Fig. 3:**
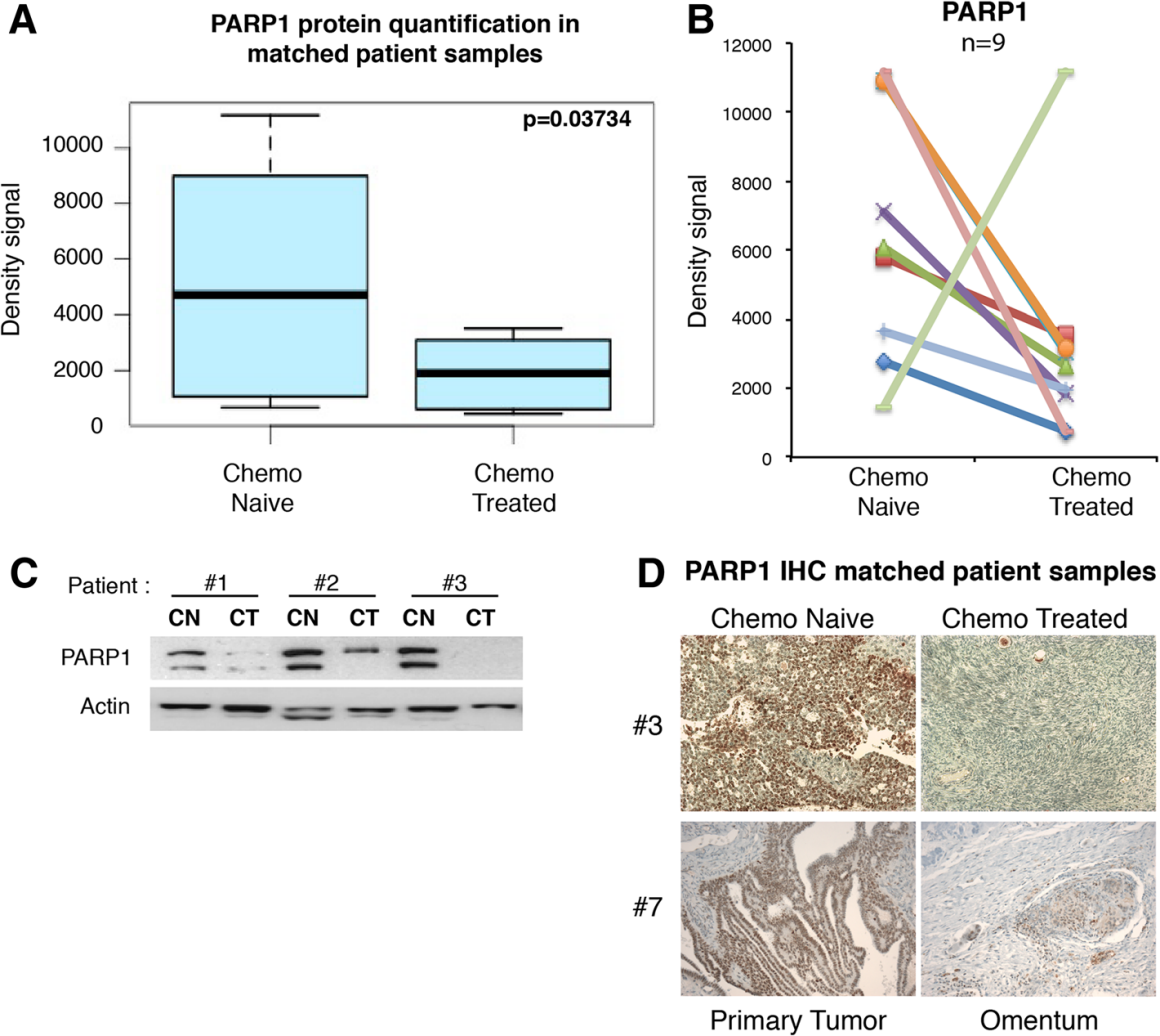
Chemotherapy decreases PARP1 protein levels in matched patient samples. **a** PARP1 protein levels in tumor samples from the same 15 patients before and after chemotherapy were quantified with ImageJ and the density signals obtained were used to generate a boxplot. Paired Mann–Whitney test *p* = 0.03734. **b** PARP1 density signals from nine patients with the presence of PARP1 in the tumors before receiving chemotherapy. **c** Representative PARP1 and actin western blot of three matched tumor samples before (*CN*) and after (*CT*) receiving chemotherapy treatment. **d** PARP1 immunohistochemistry (*IHC*) staining in tumor before and after chemotherapy treatment in the same patients (20×)

Amongst our samples, we identified 15 matched samples isolated from individual patients from whom a tumor sample was available before and after chemotherapy. Of the patients tested, six showed no PARP1 protein expression. Of the remaining nine, eight had reduced PARP1 protein levels post-chemotherapy (Fig. 3a–c). We further confirmed these results by IHC (Fig. 3d). Although surprising, our results might explain the low, or absent, PARP1 protein seen in a large proportion of the ovarian tumors tested in previous studies [18, 19]. Altogether, these data strongly suggest a suppressive effect of chemotherapy on PARP1 protein expression in solid tumors.

Next, we documented the time between the last chemotherapy treatment and the time at which the biopsy was taken in PARP1-positive and PARP1-negative tumors and found no difference (16.5 days versus 17.0 days). This suggests that the reduction of PARP1 in the tumors was stable and represents a switch in the molecular pathways of the remaining tumor cells post-chemotherapy or the selection of residual cells that have low PARP1 expression.

In addition, we evaluated whether the expression of PARP1 contributes to platinum sensitivity (defined as no tumor recurrence for greater than 6 months as per “Methods”). These data were available for the 69 tumors within our training cohort. Notably, we found that tumors with high PARP1 expression were significantly more platinum sensitive than low PARP1 expression tumors, independent of previous chemotherapy exposure in the training cohort (χ^2^ test, *p* < 0.001). This may be associated with the fact that PARP1 is an important mediator of cell death in response to stress [20].

We also confirmed using the training cohort that BRCA1-deficient tumors were associated with platinum sensitivity (χ^2^ test, *p* < 0.001). Taken together, these findings reinforce the idea that platinum-sensitive BRCA1-deficient tumors might respond better to PARP inhibitors when intratumoral PARP1 levels are high.

It is expected that PARP1 expression and a loss of functional BRCA1 are required for sensitivity to PARP inhibitors. In validation cohort 2, the BRCA1-negative, PARP1-positive subset of tumors represented 32.7 % of serous ovarian cancer patients in this study. Strikingly, this number is similar to the proportion of reported positive objective response rate in clinical trials with Olaparib (41 % [10], 33 % [8], and 25–31 % [11]). Moreover, no impact of chemotherapy on BRCA1 protein status could be discerned, emphasizing the specificity of the findings. The results suggest that patients receiving PARP inhibitors could be selected not only based on their BRCA1 status, but also for PARP1 protein expression. Such screening is commonplace for other targeted therapies, such as aromatase inhibitors or vemurafenib [21, 22], and could be integrated into standard operating procedures for pathologists and oncologists. This concept will be explored in the future within our department. Based on our data, we predict this screening approach would substantially expand the positive objective response rates.

## Conclusion

To our knowledge, this study is the first to probe the effect of chemotherapy treatment on PARP1 protein expression in HGSC. Considering that all patients enrolled in previous clinical trials received several cycles of chemotherapy prior to receiving PARP inhibitor treatment [5, 8–12], these data are likely relevant for guiding future administration of PARP inhibitor therapy. Further studies with independent cohorts are thus warranted to evaluate the effect of PARP1 protein expression on the efficacy of PARP inhibitors. These observations suggest that co-application of PARP inhibitors and chemotherapy as frontline therapy might yield significantly better outcome in HGSC, and could also be explored in other patients with homologous recombination-deficient cancers.

## Additional file

Additional file 1: Figure S1. PARP1 antibody validation. (**A**) Western blot of PARP1 and actin were performed on OVCAR8 cells infected with shRNA control or directed against PARP1. (**B**) The same cells used in (A) were paraffin-embedded and IHC staining against PARP1 was performed using the same antibody as in (**A**). The antibody shows high specificity and sensitivity for PARP1 protein in both techniques. (TIFF 1130 kb)

## Abbreviations

BRCA1/2: breast cancer 1/2
FIGO: International Federation of Gynecology and Obstetrics
HGSC: high-grade serous carcinoma
IHC: immunohistochemistry
PARP: poly(ADP)-ribose polymerase
TCGA: The Cancer Genome Atlas
