# Supplementary figures and images for: Chemotherapy reduces PARP1 in cancers of the ovary: implications for future clinical trials involving PARP inhibitors
Source: BMC Med. 2015 Sep 9;13:217. doi: 10.1186/s12916-015-0454-9 (PMC4565010; doi:10.1186/s12916-015-0454-9)

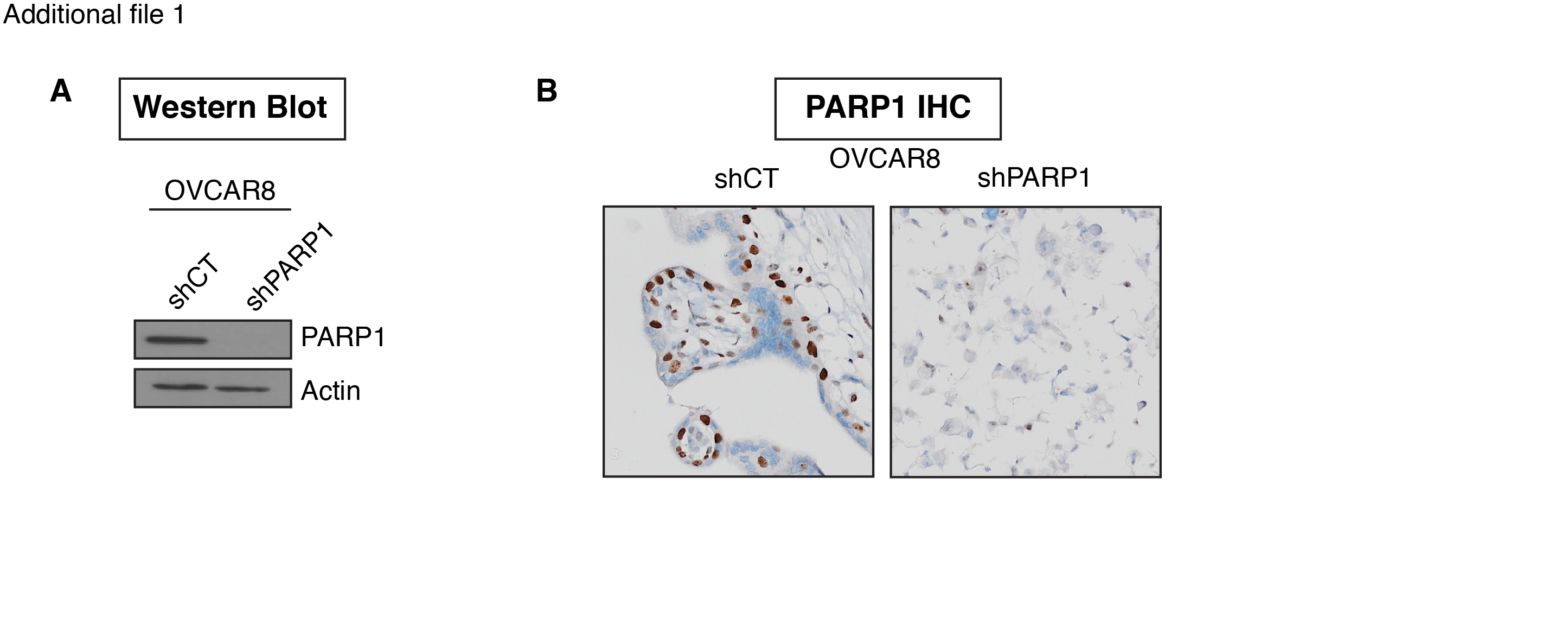

Supplement: Additional file 1: Figure S1. — PARP1 antibody validation. (A) Western blot of PARP1 and actin were performed on OVCAR8 cells infected with shRNA control or directed against PARP1. (B) The same cells used in (A) were paraffin-embedded and IHC staining against PARP1 was performed using the same antibody as in (A). The antibody shows high specificity and sensitivity for PARP1 protein in both techniques. (TIFF 1130 kb) [file 12916_2015_454_MOESM1_ESM.tif]
